# Association between blood pressure and dementia in older adults: a cross-sectional study from China

**DOI:** 10.3389/fnagi.2024.1466089

**Published:** 2024-09-11

**Authors:** Tingting Yi, Zhou Su, Jiyang Wang, Jinghuan Gan, Hao Wu, Zhihong Shi, Zhen Sun, Shuai Liu, Yong Ji

**Affiliations:** ^1^Clinical College of Neurology, Neurosurgery and Neurorehabilitation, Tianjin Medical University, Tianjin, China; ^2^Department of Neurology, Tianjin Huanhu Hospital, Tianjin Key Laboratory of Cerebrovascular and Neurodegenerative diseases, Tianjin dementia institute, Tianjin, China; ^3^Department of Neurology, The First Affiliated Hospital of Xinxiang Medical University, Xinxiang, Henan, China; ^4^Department of Neurology, People's Hospital of Qingxian, Cangzhou, China; ^5^Department of Neurology, Beijing Friendship Hospital, Capital Medical University, Beijing, China; ^6^Department of Neurology, Linfen Central Hospital, Linfen, Shanxi, China

**Keywords:** blood pressure index, dementia, diastolic blood pressure, older adults, U-shaped association

## Abstract

**Background and aims:**

The association between blood pressure (BP) and dementia in older adults remains unclear, prompting this study to investigate the relationship between various BP indicators and dementia in this population.

**Methods:**

A cross-sectional survey was conducted in 2019, including 3,599 participants aged 65 years or older. The basic demographic characteristics of participants were collected. BP measurements and neuropsychological assessments were performed. From the systolic BP (SBP) and diastolic BP (DBP) values, mean arterial pressure (MAP), pulse pressure (PP) and blood pressure index (BPI) were calculated. Generalized additive models and logistic regression models were used to analyze the association between BP indicators and dementia.

**Results:**

Generalized additive models identified a U-shaped relationship between DBP and dementia, which was more significant in males and people 70 years of age and older. The optimal DBP associated with the lowest dementia risk was 85 mmHg. Logistic regression models revealed that compared to the DBP subgroup (80–89 mmHg), participants in the DBP < 80 mmHg subgroup and the DBP ≥100 mmHg subgroup had OR for dementia of 1.611 (95% CI: 1. 252–2.073, *P* < 0.001) and 1.423 (95% CI: 0.999–2.028, *p* = 0.050), respectively. A significant association was observed between BPI and dementia (OR:1.746 95% CI: 1.142–2.668, *p* = 0.010).

**Conclusion:**

In older adults, we found a U-shaped relationship between DBP and dementia, and a linear relationship between BPI and dementia. These results underscore the importance of considering DBP and BPI in BP management strategies for older adults to potentially prevent or delay dementia onset.

## 1 Introduction

Dementia is a major cause of disability and death among people over the age of 65 globally (Li et al., 2015; GBD, 2019). Currently, more than 50 million people are living with dementia worldwide and this number is expected to exceed 150 million by 2050 (GBD, 2022), imposing a heavy burden on society and families. However, very limited treatment options are available for dementia, highlighting the importance of identifying modifiable risk factors and protective factors to reduce the incidence of this disease.

Hypertension is now recognized as one of the most common modifiable risk factors for dementia. Strong evidence suggests that the risk of all-cause dementia is increased by about 60% in middle-aged hypertension (Livingston et al., 2020). However, research on elderly populations yielded controversial results regarding the relationship between systolic blood pressure (SBP), diastolic blood pressure (DBP) and dementia. Some studies reported no association (Hebert et al., 2004; Gottesman et al., 2014; van Middelaar et al., 2018), while others found U-shaped (Lv et al., 2017) or Hockey-stick-shaped associations (Yuan et al., 2019). These inconsistent findings may be due to various factors influencing the relationship between blood pressure and dementia, such as age, sex, genetics, regional lifestyle and antihypertensive drugs (Hanon et al., 2004; Kern et al., 2017; Levine et al., 2022; Omboni et al., 2023).

As people age, SBP gradually increases and DBP gradually decreases due to decreased elastic function of large arteries and atherosclerosis (Omboni et al., 2023). Additionally, low DBP can reduce brain perfusion, increasing the risk of dementia (Lee et al., 2020; Suri et al., 2020). A prospective cohort study reported a decline in DBP in the years preceding dementia onset (Skoog et al., 1996).

Non-invasive arterial stiffness measurements, such as pulse pressure (PP) and mean arterial pressure (MAP), have long been used to assess the risk of cardiovascular events in elderly individuals (Assmann et al., 2005; Zheng et al., 2008; Hadaegh et al., 2012). Recent studies have shown that high PP increases the risk of dementia (Jung et al., 2020; Li et al., 2022), although not all studies agree (Lv et al., 2017). Other studies have suggested that MAP may be a better blood pressure predictor of dementia (Lv et al., 2017; Santillo et al., 2024). Nonetheless, further studies are required to clarify their relationship with dementia.

Following research on blood pressure, a new indicator called the blood pressure index (BPI) was developed (Ates et al., 2017a,b). One study showed that older adults with a high BPI were more likely to develop cognitive decline (Naharci and Katipoglu, 2021). The primary mechanism may be a decrease in cerebral blood flow due to a decrease in cardiac ejection volume, leading to an increased risk of cognitive impairment (Kresge et al., 2020). However, there is a relatively limited amount of data regarding the relationship between BPI and dementia among Chinese community-dwelling older adults.

In summary, this study aimed to investigate the relationship between various blood pressure indicators (SBP, DBP, MAP, PP and BPI) and dementia in older adults aged 65 and above.

## 2 Materials and methods

### 2.1 Participants

A population-based, door-to-door, cross-sectional survey was carried out in Jixian County, Tianjin, China, in 2019 and provided the data for this study. The survey covered 62 communities in Jixian County, which had similar environments and low population mobility.

**Figure 1:**
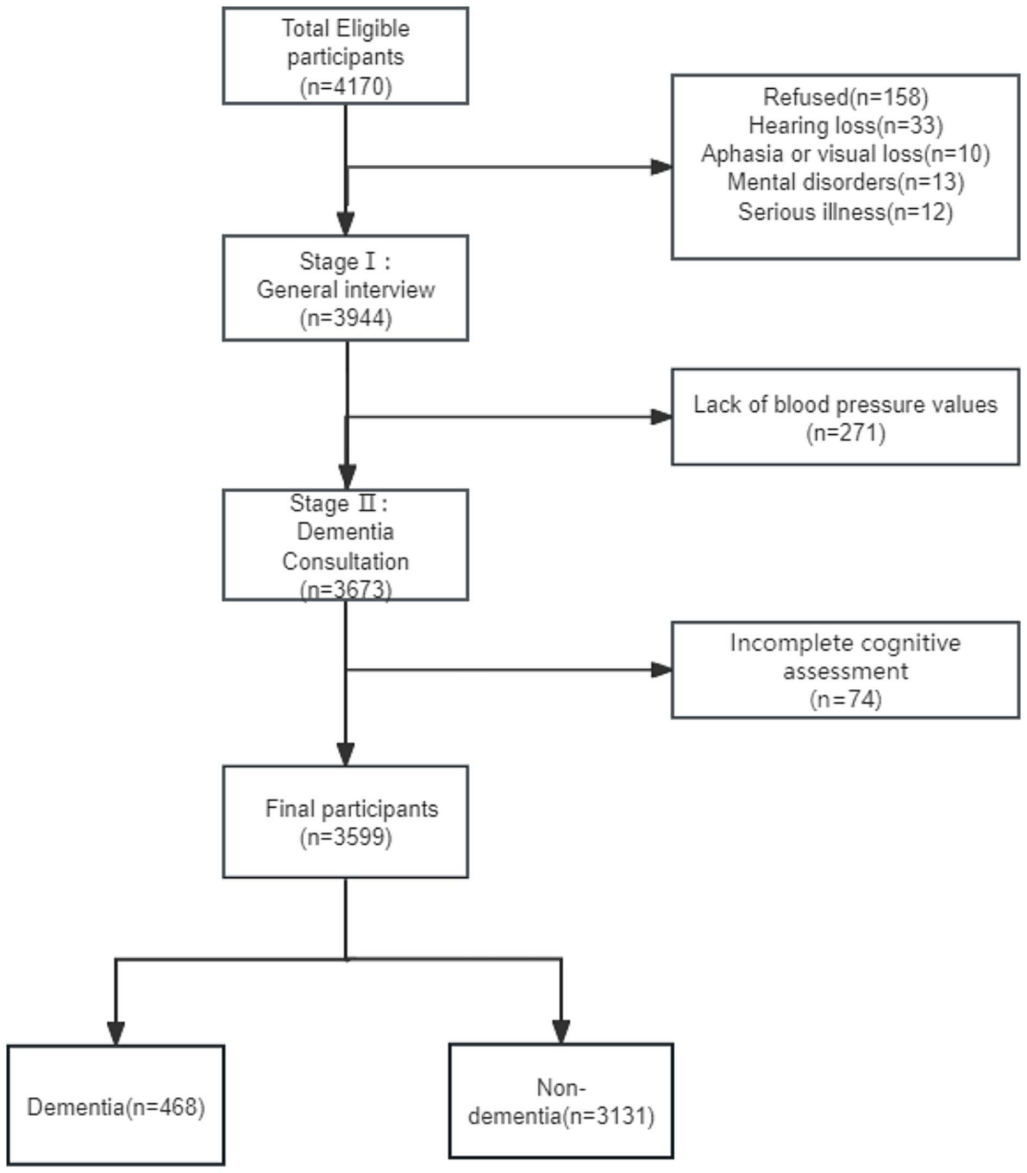
Flowchart showing how the study’s participants were chosen.

All participants included in the study were aged 65 years or older and had resided in the community for at least 5 years before the survey date. A total of 4,170 study subjects were surveyed and information was collected from 3,944 participants after excluding those with hearing loss, speech or vision loss, mental disorders, other major illnesses or refusal to participate. Participants with incomplete information were also excluded and a total of 3,599 people were included in this study (Figure 1).

The Tianjin Health Bureau and the Committee for Medical Research Ethics of Tianjin Huanhu Hospital (ID: 2019–40) approved the study protocol. Participants provided informed consent either directly or indirectly through their guardians.

### 2.2 Measures

#### 2.2.1 Stage I: general interview

At this stage, participants underwent a centralized medical examination at the local health center, where they completed a face-to-face questionnaire. During the interview process, the demographic characteristics of all participants were collected, including name, age, sex, education and marital status. Furthermore, comorbidities, such as hypertension, diabetes, heart disease and sleep disorders (Pavlova and Latreille, 2019) were recorded, and lifestyle details, such as smoking and alcohol consumption were noted. In addition, blood pressure was measured and recorded in detail by qualified and experienced neurologists, assisted by medical staff from the local health center, all of whom had received the same training at Tianjin Huanhu Hospital in China.

#### 2.2.2 Stage II: dementia consultation

At this stage, cognitive function was assessed for all participants using the Mini-Mental State Examination (MMSE) (Mitchell, 2009), the Clinical Dementia Rating Scale (CDR) (Morris, 1993) and social functioning was assessed using the Activities of Daily Living (ADL) scale (Eto et al., 1992). When MMSE test scores fall below the cut-off points (≤17 for illiterate individuals, ≤20 for those with 1–6 years of education and ≤ 24 for those with ≥7 years of education) (Zhang et al., 1990), and/or a CDR of 0.5 or higher is observed, a diagnosis of dementia is made by referencing the criteria outlined in the Diagnostic and Statistical Manual of Mental Disorders, Fourth Edition (DSM-IV) (American Psychiatric Association, 2009).

Participants were divided into two groups, namely the dementia group and the non-dementia group. The assessment was conducted by qualified and experienced neurologists who had received the same training at Tianjin Huanhu Hospital in China.

### 2.3 BP information

Participants’ blood pressure was measured between 8:00 a.m. and 10:00 a.m. using a mercury column sphygmomanometer with an appropriate cuff in the blood pressure measurement room of the health center. The participants were in a seated position with the right arm flat on a table at the same level as that of the heart and the elbow was straightened with the palm upwards. The cuff was applied to the bare upper arm, approximately 2–3 cm above the elbow crease. After resting for at least 5 min, three consecutive measurements were taken with a 1-min interval between each. The average of these three readings was calculated.

PP was calculated as the difference between SBP and DBP (Sesso et al., 2000). The MAP was calculated as (SBP + DBP × 2)/3 (Sesso et al., 2000). BPI was calculated as the SBP divided by DBP (Ates et al., 2017a,b; Naharci and Katipoglu, 2021).

### 2.4 Data analysis

Categorical variables were expressed as numbers (percentages) and continuous variables were expressed as the mean ± standard deviation (SD). The baseline characteristics of both groups were compared. The *t*-test (for normally distributed data) or the Mann–Whitney test (for non-normally distributed data) was used to compare continuous variables between groups, and the χ^2^-test was used for categorical variables. *p*-values were adjusted using the Bonferroni correction.

Subsequently, smooth curve fitting was performed using generalized additive models (GAMs) to analyze the linear or curvilinear relationships between different blood pressure indicators (SBP, DBP, MAP, PP and BPI) and dementia. The degree of freedom (EDF) of the smooth curve fitting was greater than 1, indicating a nonlinear relationship (Motulsky and Christopoulos, 2004), between DBP and MAP and dementia. The threshold effect of DBP and MAP on dementia was then analyzed using segmented regression models, the LRT test (likelihood ratio test, comparing the difference between Model I and Model II) and bootstrap resampling were used to derive the turning point of blood pressure value. Next, based on previous evidence (Lv et al., 2017; Hua et al., 2019; Yuan et al., 2019), participants were exploratively categorized into four groups (<80 mmHg, 80–89 mmHg, 90–99 mmHg, and ≥ 100 mmHg) based on DB*p* values. The patients were then stratified according to sex and age.

Finally, multivariate logistic regression was conducted to explore the association between different blood pressure indicators and dementia. In the final model, common risk factors for dementia were adjusted (Ji et al., 2015; Livingston et al., 2020; Naharci and Katipoglu, 2021), including age, sex, education, marital status, smoking, alcohol consumption, sleep disorders and stroke.

The odds ratios (ORs) and 95% confidence intervals (CIs) for the prevalence of dementia were obtained. The statistical significance of all the results was expressed by two-tailed *p* values, with *P* values less than 0.05 indicating statistical significance. The statistical analysis was carried out with Empower software and R (version 4.2.0).

## 3 Results

### 3.1 Baseline characteristics

This study included 3,599 individuals, 13.00% (*n* = 468) of whom had dementia (Figure 1). The mean age of the participants was 72.02 ± 5.77 years and 55.77% were females.

Table 1 displays the demographic characteristics, comorbidities, and various blood pressure indicators of the sample. Older females, those who were less educated, and unmarried or widowed were more prone to develop dementia (*p* < 0.001). The dementia group showed a greater prevalence of hypertension, stroke and sleep disorders compared to the non-dementia group (*p* < 0.05). No statistically significant differences were found between the two groups in body mass index, smoking, diabetes, heart disease or orthostatic hypotension (*p* > 0.05).

**Table 1:** Characteristics of participants.

Variables	Total (*n* = 3,599)	Nondementia (*n* = 3,131)	Dementia (*n* = 468)	*P-*value
Age at interview, mean ± SD, years	72.02 ± 5.77	71.60 ± 5.41	74.84 ± 7.12	<0.001^***^
65–69	1,498 (41.62)	1,361 (43.47)	137 (29.27)	
≥70	2,101 (58.38)	1770 (56.53)	331 (70.73)	
Female gender,n (%)	2007 (55.77)	1,670 (53.34)	337 (72.01)	<0.001^***^
Education (years),n (%)				<0.001^***^
<1	994 (27.62)	798 (25.49)	196 (41.88)	
1–6	1911 (53.10)	1,694 (54.10)	217 (46.37)	
>6	694 (19.28)	639 (20.41)	55 (11.75)	
BMI, mean ± SD	25.72 ± 3.99	25.74 ± 3.94	25.58 ± 4.36	0.144
Marital status,n (%)				<0.001^***^
Single	37 (1.03)	28 (0.89)	9 (1.92)	
Married	2,715 (75.44)	2,414 (77.10)	301 (64.32)	
Divorced	33 (0.92)	29 (0.93)	4 (0.85)	
Widowed	814 (22.62)	660 (21.08)	154 (32.91)	
Smoking,n (%)	716 (19.89)	619 (19.77)	97 (20.73)	0.629
Alcohol consumption,n (%)	1,031 (28.65)	940 (30.02)	91 (19.44)	<0.001^***^
Comorbidity				
Hypertension,n (%)	2038 (56.63)	1748 (55.83)	290 (61.97)	0.012^*^
DM,n (%)	494 (13.73)	418 (13.35)	76 (16.24)	0.090
Stroke,n (%)	479 (13.31)	373 (11.91)	106 (22.65)	<0.001^***^
Heart disease,n (%)	619 (17.20)	528 (16.86)	91 (19.44)	0.168
OH,n (%)	421 (11.70)	356 (11.37)	65 (13.89)	0.114
Sleep disorders,n (%)	881 (24.48)	743 (23.73)	138 (29.49)	0.007^**^
Blood pressure measurements				
SBP mmHg, mean ± SD	151.71 ± 21.62	151.50 ± 21.41	153.14 ± 22.96	0.319
DBP mmHg, mean ± SD	85.31 ± 11.22	85.55 ± 11.00	83.64 ± 12.49	<0.001^***^
PP mmHg, mean ± SD	66.41 ± 17.98	65.95 ± 17.87	69.49 ± 18.42	<0.001^***^
MAP mmHg, mean ± SD	107.44 ± 12.96	107.54 ± 12.75	106.81 ± 14.29	0.160
BPI, mean ± SD	1.79 ± 0.23	1.78 ± 0.23	1.85 ± 0.24	<0.001^***^

^*^p < 0.05, ^**^p < 0.01, ^***^p < 0.001.P means the comparison between “Nondementia” and “Dementia.”BMI, body mass index; DM, diabetes mellitus; OH, orthostatic hypotension; SBP, systolic blood pressure; DBP, diastolic blood pressure; MAP, mean arterial pressure; PP, pulse pressure; BPI, blood pressure index. Num, number.

In this study, the overall prevalence of hypertension was 56.63%. Compared to the non-dementia group, the individuals in the dementia group exhibited a greater prevalence of hypertension (*p* < 0.05), with lower DBP (DBP 83.64 ± 12.49 vs. 85.55 ± 11.00, *p* < 0.001), but higher PP and BPI (PP 69.49 ± 18.42 vs. 65.95 ± 17.87, *p* < 0.001 and BPI 1.85 ± 0.24 vs. 1.78 ± 0.23, *p* < 0.001). However, no statistically significant difference was detected in SBP or MAP between the two groups (*p* > 0.05).

### 3.2 Nonlinear associations between various blood pressure indicators and dementia

**Figure 2:**
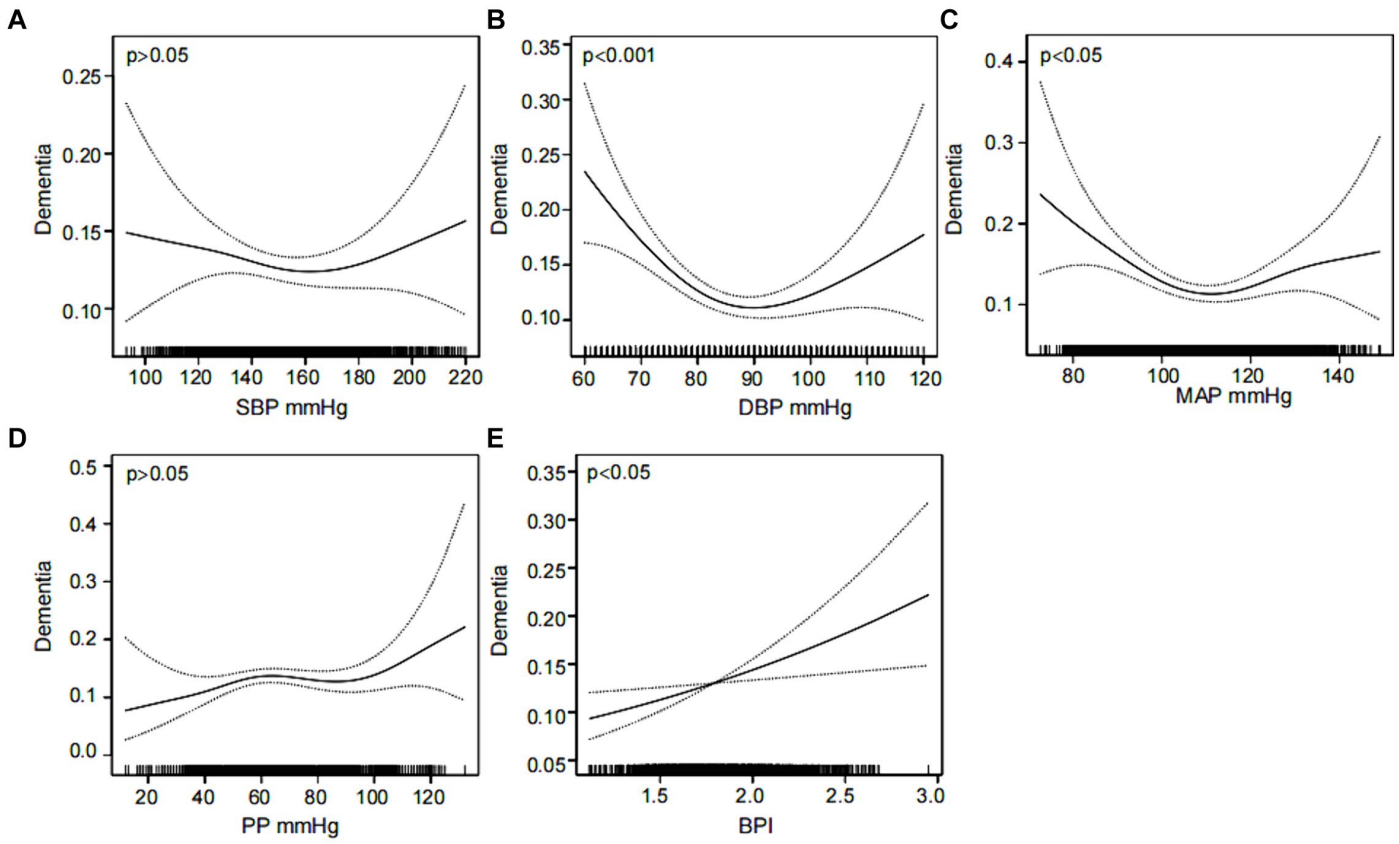
Nonlinear associations between various blood pressure indicators and dementia. The association of SBP **(A)**, DBP **(B)**, MAP **(C)**, PP **(D)** and BPI **(E)** with the prevalence of dementia was analyzed using a smooth curve fitting method, respectively. The adjustment factors included sex, age, education, marital status, smoking, alcohol consumption, sleep disorders and stroke. The solid black line in the middle is the fitted smoothed curve, and the grey dashed lines on both sides are the 95% confidence intervals. SBP, systolic blood pressure; DBP, diastolic blood pressure; MAP, mean arterial pressure; PP, pulse pressure; BPI, blood pressure index.

Furthermore, the association between dementia and SBP, DBP, MAP, PP and BPI was investigated separately using smoothed curve fitting. The results adjusted for confounders are displayed in Figure 2. A U-shaped relationship was observed between DBP and MAP and dementia (EDF = 2.50 and 2.80, *p* < 0.001 and *p* = 0.007, respectively) (Figures 2B,C). In addition, a linear association was found between BPI and dementia (EDF = 1.00, *p* = 0.010) (Figure 2E). However, the associations between SBP and PP and dementia were not significant (*p* > 0.05) (Figures 2A,D).

Segmented regression models were used to ascertain the threshold effect of DBP and MAP on dementia. The findings are displayed in Table 2. After adjusting for confounders, the values of DBP and MAP corresponding to the turning points were 85 mmHg and 109.33 mmHg, respectively. Before the turning point, both DBP and MAP were significantly associated with dementia. The odds ratios (ORs) were 0.956 (95% CI: 0.940–0.973, *p* < 0.001) for DBP and 0.972 (95% CI: 0.959–0.986, *p* < 0.001) for MAP. After the turning point, the associations remained significant, with ORs of 1.020 (95% CI: 1.003–1.037, *p* = 0.018) for DBP and 1.019 (95% CI: 1.004–1.035, *p* = 0.014) for MAP.

**Table 2:** The threshold effect of DBP and MAP on dementia was analyzed using segmented regression model.

Outcome	DBP		MAP	
	Adjusted model OR (95% CI)	*P*-value	Adjusted model OR (95% CI)	*P*-value
Model I	0.988(0.980, 0.997)	0.012^∗^	0.994(0.986, 1.001)	0.110
Model II				
Turning point (mmHg)	85		109.33	
≤Turning point	0.956 (0.940, 0.973)	<0.001^***^	0.972(0.959, 0.986)	<0.001^***^
>Turning point	1.020 (1.003, 1.037)	0.018^*^	1.019(1.004, 1.035)	0.014^∗^
LRT test	<0.001^#^		<0.001^#^	

^*^P < 0.05, ^**^P < 0.01, ^***^P < 0.001; Model I, linear analysis; Model II, non-linear analysis.^#^means Model II is significantly different from Model I, which indicates a non-linear relationship. Adjusted for age, sex, education, marital status, smoking, alcohol consumption, sleep disorders and stroke.DBP, diastolic blood pressure; MAP, mean arterial pressure; OR, odds ratio; CI, confidence interval; LRT test, Logarithmic likelihood ratio test.

### 3.3 Subgroup analysis of the association between DBP and dementia

**Figure 3:**
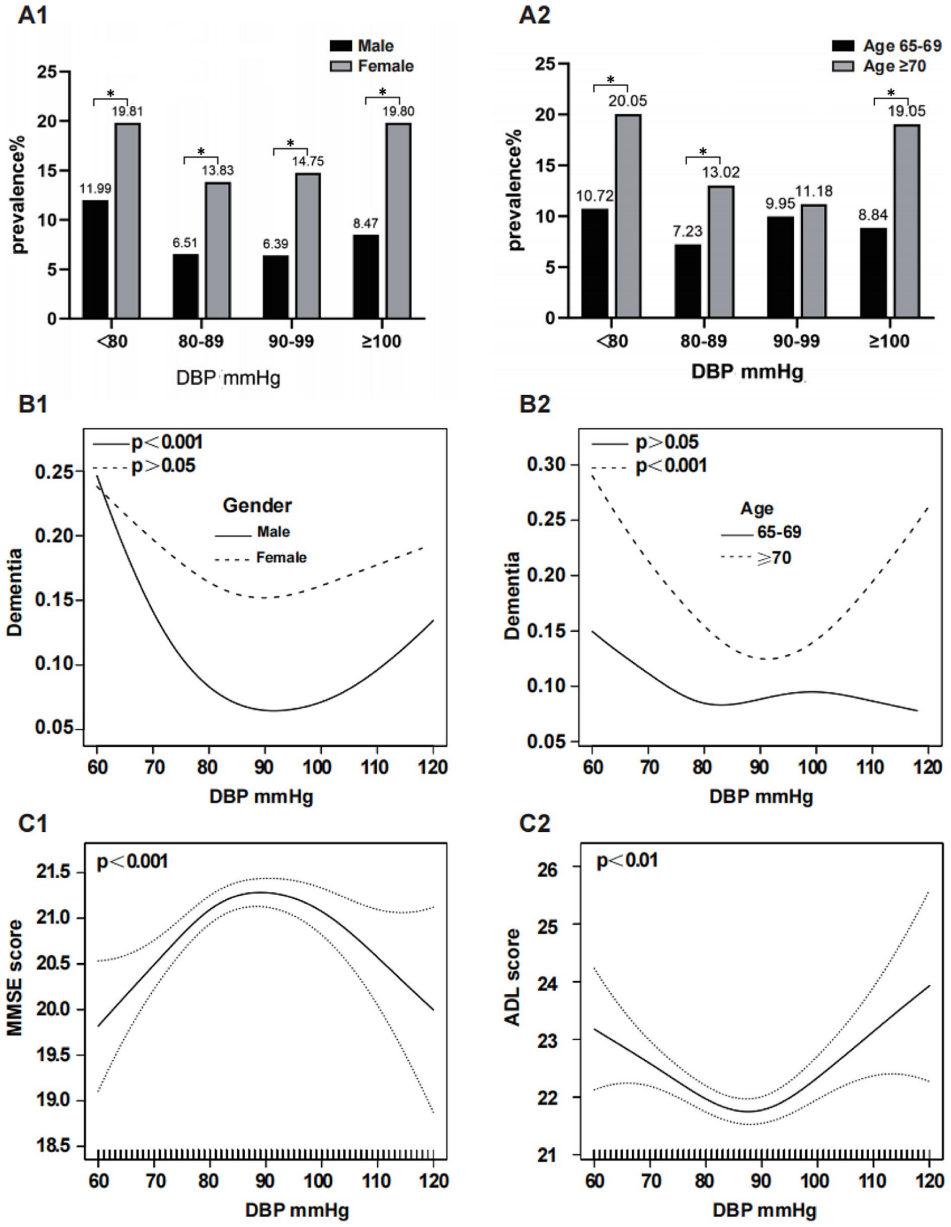
Subgroup analysis of the association between DBP and dementia. The prevalence of dementia in the DBP subgroups stratified by sex and age is shown in **(A1,A2)**. The association between DBP and dementia, MMSE score and ADL score was analyzed by smoothed curve fitting: **(B1)** was stratified by sex and adjusted for age, education, marital status, smoking, drinking, sleep disorders and stroke; **(B2)** was stratified by age and adjusted for sex, education, marital status, smoking, drinking, sleep disorders and stroke; **(C1,C2)** adjusted for age, sex, education, marital status, smoking, alcohol consumption, sleep disorders and stroke, respectively; ^*^means *p* < 0.05. DBP, Diastolic Blood Pressure.

Figure 3 illustrates the relationship between DBP and dementia, stratified by sex and age. A subgroup analysis by sex revealed a significantly higher prevalence of dementia in females compared to males within each subgroup: 19.81% vs. 11.99, 13.83% vs. 6.51, 14.75% vs. 6.39, and 19.80% vs. 8.47% (*p* < 0.05) (Figure 3A1). After adjusting for confounders, smoothed curve fitting showed a U-shaped relationship between DBP and dementia in males (EDF = 2. 39, *p* < 0.001), with the turning point being 87 mmHg; however, no significant association was observed between DBP and dementia in females (EDF = 2.08, *p* = 0.08) (Figure 3B1). In all the subgroups, the prevalence of dementia was greater in the ≥70 years age group than in the 65–69 years age group: 20.05% vs. 10.72%, *p* < 0.05; 13.02% vs. 7.32%, *p* < 0.05; 11.18% vs. 9.95%, *p* > 0.05; 19.05% vs. 8.84%, *p* < 0.05 (Figure 3A2). After adjusting for confounders, DBP exhibited a U-shaped relationship with dementia at age ≥ 70 years (EDF = 2.72, *p* < 0.001), with the turning point being 90 mmHg; however, this relationship was not significant at age 65–69 years (EDF = 2.62, *p* = 0.469) (Figure 3B2). DBP had an inverted U-shaped correlation with the MMSE score (EDF = 2.65, *p* < 0.001) (Figure 3C1) and a U-shaped relationship with the ADL score (EDF = 2.69, *p* = 0.003) after adjusting for confounders. (Figure 3C2).

### 3.4 Association between blood pressure and dementia

Multivariate logistic regression analyses of the association between various blood pressure indicators and dementia were performed, and the results are shown in Table 3.

**Table 3:** Associations between blood pressure classification and dementia according to the various models.

Variables	ModelI OR (95% CI)	*P-*value	Model II OR (95% CI)	*P-*value	Model III OR (95% CI)	*P-*value	Model IV OR (95% CI)	*P-*value
SBP mmHg	1.003 (0.999, 1.008)	0.127	1.001 (0.996, 1.005)	0.806	1.000 (0.996, 1.005)	0.926	0.999 (0.995, 1.004)	0.790
DBP mmHg								
<80	1.719(1.346, 2.194)	<0.001^***^	1.580 (1.232, 2.027)	<0.001^***^	1.567 (1.221, 2.012)	<0.001^***^	1.611(1.252, 2.073)	<0.001^***^
80–89	1.00 reference		1.00 reference		1.00 reference		1.00 reference	
90–99	1.007 (0.758, 1.336)	0.965	1.080 (0.810, 1.441)	0.599	1.068 (0.800, 1.426)	0.655	1.075 (0.803, 1.440)	0.626
≥100	1.419 (1.009, 1.995)	0.044^*^	1.491 (1.053, 2.110)	0.024^*^	1.499 (1.058, 2.123)	0.023^*^	1.423(0.999, 2.028)	0.050^*^
Hypertension	1.289 (1.056, 1.574)	0.013^*^	1.210 (0.987, 1.483)	0.067	1.226 (0.999, 1.505)	0.051	1.114 (0.904, 1.373)	0.311
MAP mmHg	0.996 (0.988, 1.003)	0.256	0.996 (0.988, 1.003)	0.275	0.996 (0.988, 1.003)	0.254	0.994 (0.986, 1.001)	0.110
PP mmHg	1.011 (1.005, 1.016)	<0.001^***^	1.005 (0.999, 1.010)	0.100	1.004 (0.999, 1.010)	0.153	1.004 (0.998, 1.009)	0.203
BPI	3.015 (2.028, 4.483)	<0.001^***^	1.785 (1.177, 2.708)	0.006^**^	1.716 (1.127, 2.614)	0.012^*^	1.746 (1.142, 2.668)	0.010^*^

^*^P < 0.05, ^**^P < 0.01, ^***^P < 0.001.Model 1, unadjusted; Model 2, adjusted for age, sex and education; Model 3, adjusted for age, sex, education and marital status; and Model 4, adjusted for age, sex, education, marital status, smoking, alcohol consumption, sleep disorders and stroke.SBP, systolic blood pressure; DBP, diastolic blood pressure; MAP, mean arterial pressure; PP, pulse pressure; BPI, blood pressure index; OR, odds ratio; CI, confidence interval.

In the final model controlling for potential confounding factors, compared to the DBP subgroup (80–89 mmHg), participants in the DBP < 80 mmHg subgroup and the DBP ≥100 mmHg subgroup had ORs for dementia of 1.611 (95% CI: 1. 252–2.073, *P* < 0.001) and 1.423 (95% CI: 0.999–2.028, *p* = 0.050), respectively. However, in the DBP (90–99 mmHg) subgroup, the difference was not statistically significant (*p* > 0.05). In the final model, a significant association between BPI and dementia was observed (OR:1.746 95% CI: 1.142–2.668, *p* = 0.010). In the unadjusted model, hypertension and PP were significantly associated with dementia (OR: 1.289 95% CI: 1.056–1.574, *p* = 0.013; OR: 1.011 95% CI: 1.005–1.016, *P* < 0.001, respectively). However, in the adjusted final model, hypertension, SBP, PP, and MAP showed no significant association with dementia (*p* > 0.05).

## 4 Discussion

In a cross-sectional study of 3,599 Chinese community-dwelling older adults aged 65 years and older, this study revealed a U-shaped relationship between DBP and dementia, which was particularly significant for male and older adults aged 70 years and older. In addition, there was a linear relationship between BPI and dementia.

The effect of blood pressure on dementia in older adults is controversial (Hebert et al., 2004; Lv et al., 2017; Ou et al., 2020; Badji et al., 2021) and most studies have focused primarily on SBP, with less attention to DBP. The present study revealed a U-shaped correlation between DBP and dementia, consistent with several previous studies (Lee et al., 2022; van Dalen et al., 2022). In this study, with dementia risk being lowest at 85 mmHg DBP, the findings align with those of Lv et al. (2017). Moreover, the optimal range for DBP control in older adults is 80–99 mmHg, which is similar to the conclusion of 90–100 mmHg from a prospective systematic review (Ou et al., 2020). However, the study by Kumar B. Rajan et al. reported that each 5 mmHg increase in DBP above 76 mmHg was linked to a 57% greater risk of developing Alzheimer’s disease (AD) (Rajan et al., 2018). Specific optimal protective DBP values require further longitudinal studies. Nonetheless, it is undeniable that DBP plays an important role in controlling blood pressure in elderly people, and lower blood pressure does not necessarily equate to improved health. The U-shaped relationship between DBP and dementia identified in this study was more pronounced in adults aged 70 years and older. A prospective study in the Bronx Ageing Study reached a similar conclusion, suggesting that low DBP was associated with the risk of dementia in people aged 75 and older (Verghese et al., 2003). Therefore, in older adults (>70 years) with isolated systolic hypertension who already have a low DBP, antihypertensive therapy may be administered with more caution (Angeli et al., 2020; Rivasi et al., 2023).

Our findings also revealed a sex difference in the U-shaped relationship between DBP and dementia, showing a significant association only in males. This finding is in agreement with the conclusions of several previous studies (Gao et al., 2021; Gong et al., 2021). Possible reasons for this result are the differences in sex chromosomes, sex hormones, environmental factors, and lifestyle habits between males and females, which contribute to differences in the physiological and pathological processes that regulate blood pressure in males and females (Gerdts et al., 2022). Little attention has been given to sex differences in the relationship between DPB and dementia in previous studies. Therefore, further investigations are required to confirm these possible sex differences and to identify the underlying mechanisms.

BPI is a new index obtained by dividing SBP by DBP, which is mainly used to reflect heart failure and right heart insufficiency in clinical practice (Ates et al., 2017a,b). Less attention has been paid to the relationship between this parameter and dementia among Chinese community-dwelling older adults. In the present study, BPI was found to be associated with dementia in older adults. The main mechanism may be attributed to reduced cerebral blood flow due to decreased cardiac ejection, causing an increased risk of dementia (Kresge et al., 2020). In addition, a high BPI indicates a severe degree of arterial stiffness and atherosclerosis, thus increasing the risk of dementia (van Popele et al., 2001). Previous studies have reported that dementia and its two main subtypes, AD and vascular dementia, are both associated with atherosclerosis, with the etiology of AD involving the interaction between ApoE and atherosclerosis (Hofman et al., 1997; van Oijen et al., 2007). Thus, from a biological point of view, SBP increases with age, while DBP decreases, resulting in an increase in BPI, which can be linked to dementia in later life through pathologic changes (van Popele et al., 2001; Kresge et al., 2020; Omboni et al., 2023). In addition, the relationship between BPI and dementia found in this study may help to understand the association between cardiovascular risk factors and dementia in later life.

Some studies have shown that MAP is associated with an increased risk of dementia (Lv et al., 2017; Santillo et al., 2024). However, a study by Cui et al. with a 15-year follow-up revealed that MAP was not associated with an increased risk of dementia (Cui et al., 2018). In the present study, a U-shaped relationship was found between MAP and dementia in GAMs and segmented regression model; nonetheless, additional logistic regressions did not support this. In addition, a cohort study found that older adults with higher levels of SBP had a lower risk of dementia and a more pronounced U-shaped relationship was observed between SBP and the risk of dementia in older adults over the age of 75 (van Dalen et al., 2022). Moreover, high PP was found to increase the risk of dementia (Jung et al., 2020; Li et al., 2022). However, in our study, a nonlinear trend was found between SBP and PP and dementia, although the findings were not statistically significant. These inconsistencies may result from different study designs, assessment methods, sample sizes, adjustments for potential confounders, and participant characteristics such as age, sex, and ethnicity (Hanon et al., 2004; Levine et al., 2022; Omboni et al., 2023). Therefore, further research is still required.

At present, the mechanisms underlying dementia due to hypertension or hypotension are incompletely understood and may include the following. First, chronic hypertension can cause atherosclerosis, which leads to vascular remodeling, small vessel occlusion and microvascular damage, resulting in white matter lesions, perivascular gap enlargement, cerebral microhemorrhage and lacunar cerebral infarction (Connelly et al., 2005; Santisteban et al., 2023). Second, hypertension impairs the ability of vascular endothelial cells to regulate cerebral blood flow, with sudden falls in blood pressure resulting in insufficient cerebral perfusion (Iadecola and Gottesman, 2019); in addition, hypertension damages vascular endothelial cells, which leads to blood–brain barrier dysfunction under the combined effect of perivascular macrophages (Santisteban et al., 2020). Furthermore, high blood pressure can affect neurodegenerative disease by impairing vascular integrity, which can result in cerebral amyloid angiopathy, reduced brain clearance of Aβ (Shah et al., 2012) and increased levels of phosphorylated tau protein (Hu et al., 2022). Finally, hypertension can cause abnormalities in the renin-angiotensin-aldosterone system, leading to dementia (Hajjar et al., 2015).

The mechanisms of hypotension may involve inadequate cerebral perfusion, impaired nutrient delivery, impaired waste removal (Suri et al., 2020), and slowed circulation. Distal microvascular ischemia may further lead to neural tissue damage and increase the risk of dementia (Lee et al., 2020). Additionally, the interaction between the APOEε4 allele and low DBP can further elevate the risk of developing AD (Qiu et al., 2003). In late life, low DBP may result in insufficient cerebral perfusion, leading to a chronic hypoperfusion state. This condition may induce or exacerbate abnormal tau protein phosphorylation. Persistent hypoperfusion can cause inadequate oxygen and nutrient supply to brain tissue, promoting neuroinflammation and oxidative stress. These processes may further lead to the abnormal accumulation of tau protein and the formation of neurofibrillary tangles, impairing neuronal function and synaptic connectivity, ultimately leading to the development of dementia (Hu et al., 2022). Furthermore, low DBP often indicates arterial stiffness and loss of elasticity, which are typically manifestations of atherosclerosis. Vascular stiffness caused by atherosclerosis may restrict blood flow to the brain, particularly during diastole when the heart is at rest, limiting the brain’s ability to receive blood through hardened arteries. This reduction in blood flow may lead to chronic cerebral ischemia, especially in vulnerable regions of the brain, such as those involved in memory and cognitive function. These regions, when subjected to prolonged hypoxia and nutrient deprivation, may experience progressive neuronal loss, ultimately leading to the development of dementia (Bots et al., 1996).

The strengths of this study are as follows. First, a large-scale survey of Chinese community members aged 65 years or older was performed. The nonlinear relationship between various blood pressure indicators and dementia was assessed, with a focus on DBP and the new index of blood pressure, BPI. Finally, differences in sex and age were explored.

Nevertheless, the limitations of the present study should be acknowledged. First, since the study was cross-sectional, a causal link between DBP and dementia could not be established. Second, in the blood pressure measurements, 24-h ambulatory blood pressure monitoring was not performed and possible daily changes (circadian rhythms, general physiologic variability, etc) could not be examined. Third, even after adjusting for possible confounders, other factors, such as the APOE-epsilon4 allele, psychological stress, diet and other changes may impact the results. Finally, the participants included in this study were all elderly people in the Tianjin community, so caution is needed when generalizing the results to the entire elderly population.

## 5 Conclusion

The present study demonstrated a U-shaped association between DBP and dementia in older adults, particularly pronounced in males and those aged 70 and above. Additionally, a linear association was observed between BPI and dementia in this population. These findings suggest that DBP and BPI are critical factors to consider when counseling older adults on blood pressure management, as they may play a key role in preventing or delaying the onset of dementia. Future prospective cohort studies are needed to confirm these associations.

## Data Availability

The original contributions presented in the study are included in the article material, further inquiries can be directed to the corresponding authors.
